# Freezing and unfreezing of antiferromagnetic spins in CoO(111) epitaxial films on a ferromagnetic support

**DOI:** 10.1038/s41598-025-32592-9

**Published:** 2025-12-19

**Authors:** A. Kwiatkowski, M. Szpytma, E. Świerkosz, E. Oleś, P. Dróżdż, A. Kozioł-Rachwał, M. Zając, E. Partyka-Jankowska, T. Ślęzak, M. Ślęzak

**Affiliations:** 1 AGH University of Krakow, Kraków, Poland; 2 Elettra-Sincrotrone Trieste, Trieste, Italy; 3 SOLARIS National Synchrotron Radiation Centre, Jagiellonian University, Kraków, Poland

**Keywords:** Materials science, Physics

## Abstract

Epitaxial CoO(111)/Fe(110) bilayers grown on W(110) single crystal were studied using magneto-optic Kerr effect and X-ray magnetic linear dichroism techniques. A variety of possible configurations of frozen antiferromagnetic spins and in-plane easy axis of ferromagnet in CoO(111)/Fe(110) bilayers is presented. Our study shows that the blocking temperature, which marks the onset of exchange bias effect, strongly depends on the thickness of CoO layer. We confirmed that this dependence can be explained with the thermal stability of rotatable antiferromagnetic moments, that goes beyond classical finite-size effects. Partial unfreezing of antiferromagnetic spins at the vicinity of blocking temperature can be employed to reorient rotatable antiferromagnetic spins and refreeze them in a new orientation after subsequent cooling down the system.

**Supplementary Information:**

The online version contains supplementary material available at 10.1038/s41598-025-32592-9.

## Introduction

The exchange bias (EB) effect was discovered in 1956^1,2^ however first exchange biased system were engineered by nature a few billion years ago in titanohematite^3^. Nevertheless, the EB phenomenon in ferromagnet/antiferromagnet (FM/AFM) bilayers, still remains one of the most complex and fascinating effects that have ever been studied in the field of magnetism. It usually manifests as an enhancement of coercivity and a shift of the hysteresis loop along the field axis when the AFM/FM system is cooled through its blocking temperature (T_B_), typically but not necessarily in the presence of a magnetic field. Blocking temperature can be either identical or lower than the Néel temperature (T_N_)^4^ of the AFM layer, above which antiferromagnetic order disappears. Such unidirectional anisotropy in FM/AFM systems originates from the interfacial exchange coupling between the ferromagnetic and antiferromagnetic layers and plays a critical role in stabilizing magnetic configurations in devices such as magnetic tunnel junctions, spin valves, MRAMs and more recently also in field-free, spin–orbit torque (SOT) architectures. With this respect, the possibility to control the orientation of antiferromagnetic moments frozen^5^ below the blocking temperature of AFM/FM and the issue of their thermal stability and homogeneity, is of significant importance. In the first case, EB enabled SOT switching without the assistance of external magnetic field, as was previously demonstrated in MnPt/FM and IrMn/FM stacks^6^. The second issue relates to neuromorphic applications and artificial neural networks idea^7^. It was shown that due to inhomogeneity of EB at the AFM/FM interface the threshold currents varied leading to a change of the switching mode from binary to multilevel, memristive one which is advantageous for the realization of antiferromagnet/ferromagnet synaptic weights idea^4^.

Epitaxial AFM/FM bilayers provide a convenient platform to investigate interface-driven phenomena in a controlled manner. Unlike polycrystalline systems, epitaxial structures offer well-defined interfaces, enabling the disentanglement of intrinsic effects from extrinsic grain-boundary-related behaviour. In this respect, the Fe(110) surface provides a well-defined substrate for CoO growth and has a known in-plane magnetic anisotropy that makes it highly sensitive to exchange-induced modifications of system magnetic properties. In this context, the epitaxial CoO(111)/Fe(110) bilayer grown on W(110) presents an ideal model system for studying the fundamentals of exchange bias, in particular how antiferromagnetic order and interface coupling evolve with temperature and magnetic layers thickness. In this report, we present the variety of possible configurations for relative orientation of frozen antiferromagnetic spins and in-plane easy axis of ferromagnet in CoO(111)/Fe(110) bilayers. We show that the T_N_ of the AFM and the T_B_ temperatures do not coincide in studied system. The later one strongly depends on the thickness of CoO layer which can be explained by the thermal stability of rotatable antiferromagnetic moments that goes beyond classical finite-size effects. Unravelling these subtleties is not only of fundamental interest but may also lead to new, thermally tuneable magnetic functionalities for spintronic devices.

## Results and discussion

**Fig. 1:**
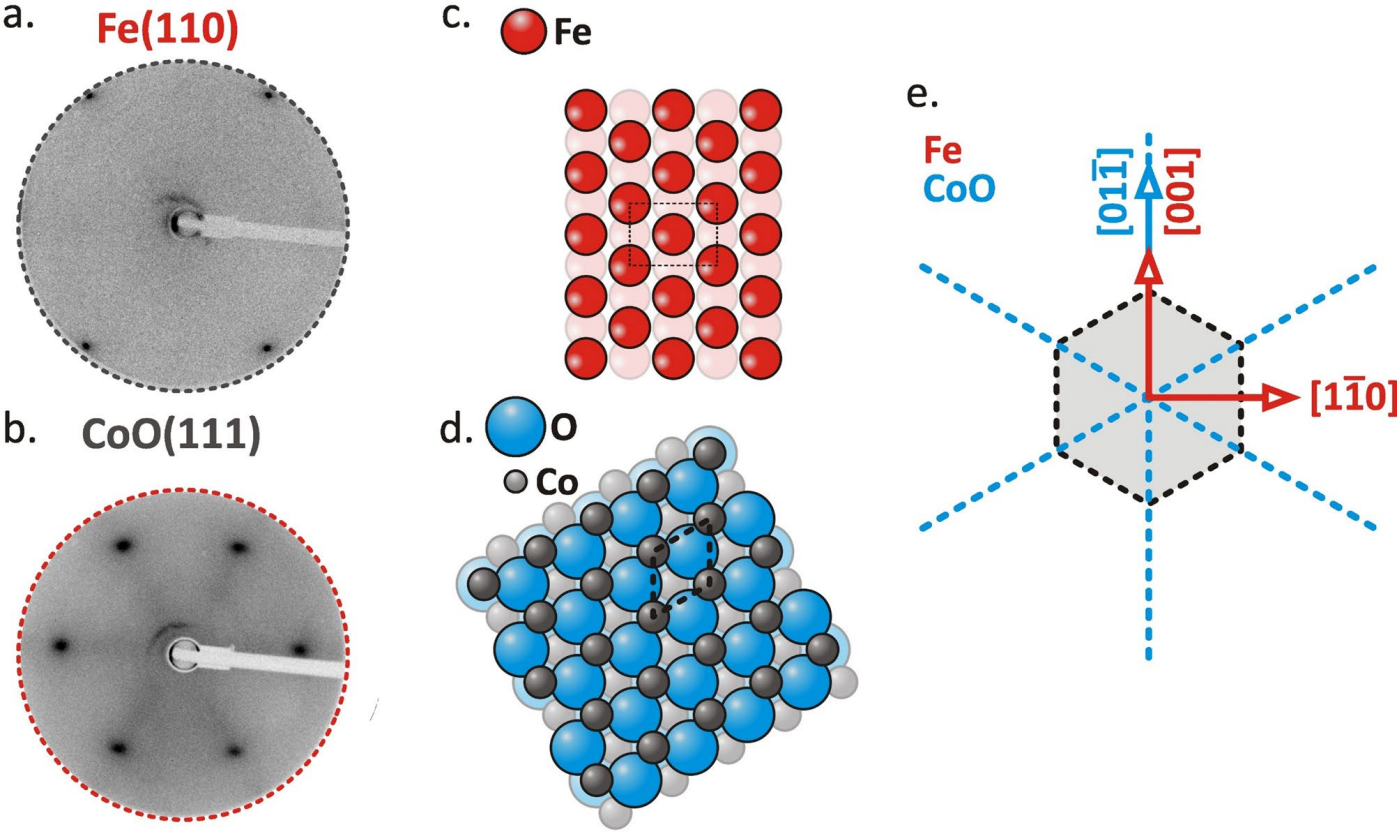
LEED patterns of (**a**) uncovered Fe(110) and (**b**) CoO(111)/Fe(110) surfaces. Corresponding ball models of (**c**) Fe(110) and (**d**) CoO(111)/Fe(110) surfaces. The relative Fe and CoO in-plane directions as concluded from the LEED analysis are sketched in (**e**). The thicknesses of Fe and CoO layers are 90 Å and 30 Å, respectively.

The typical LEED pattern of Fe grown on the W(110) surface is shown in Fig. 1a, where sharp diffraction spots clearly indicate a smooth unreconstructed Fe(110) surface. Within the 50–200 Å thickness range of Fe, we find that the in-plane lattice spacing a_001_ along the Fe[001] direction is a_001_ = 2.88 ± 0.02 Å. This matches well the value for bulk Fe and thus corresponds to an almost relaxed Fe(110)/W film. The LEED pattern from the CoO/Fe(110)/W(110) surface, shown in Fig. 1b, indicates a hexagonal CoO(111) surface structure independently from the thickness of the underlying Fe layer. Accordingly, ball models of Fe(110) and CoO(111)/Fe(110) surfaces are presented in Fig. 1c and d, respectively. Given their respective lattice parameters, CoO(111) is expected to grow epitaxially on single-crystalline Fe(110) films. In the context of a hard sphere model, the nature of epitaxial alignment can be predicted by comparing the in-plane nearest neighbour distances of the two materials, expressed as the ratio α = *d*_bcc_/*d*_fcc_. For 0.88 < α < 0.96, the so called Kurdjumov–Sachs (KS) orientation relationship is typically favoured, in which the [1̅10]_fcc_ direction in the fcc lattice aligns with the [1̅11]_bcc_ direction in the bcc lattice. Alternatively, for 0.83 < α < 0.88 or 1.02 < α < 1.19, the Nishiyama–Wassermann (NW) orientation is more likely, characterized by the alignment of [1̅10]_fcc_ with [001]_bcc_ directions. Using the nearest neighbour distances *d*_Fe_ = √3/2 × *a*_Fe_ = 2.48 Å and *d*_CoO_ = 1/√2 × *a*_CoO_ = 3.01 Å, the calculated ratio α = *d*_Fe_ / *d*_CoO_ = 0.83 suggests that CoO(111) is expected to grow on Fe(110) in the Nishiyama–Wassermann orientation^8^. This prediction is experimentally confirmed by LEED analysis presented in Fig. 1e, where [1̅10]_fcc_ || [001]_bcc_ relation is concluded. Within the studied range, the symmetry of the observed LEED patterns is independent on the thicknesses of both Fe and CoO layers. In case of CoO overlayers, for higher thicknesses, diffraction spots become slightly broadened indicating increased roughness at the CoO surface.

**Fig. 2:**
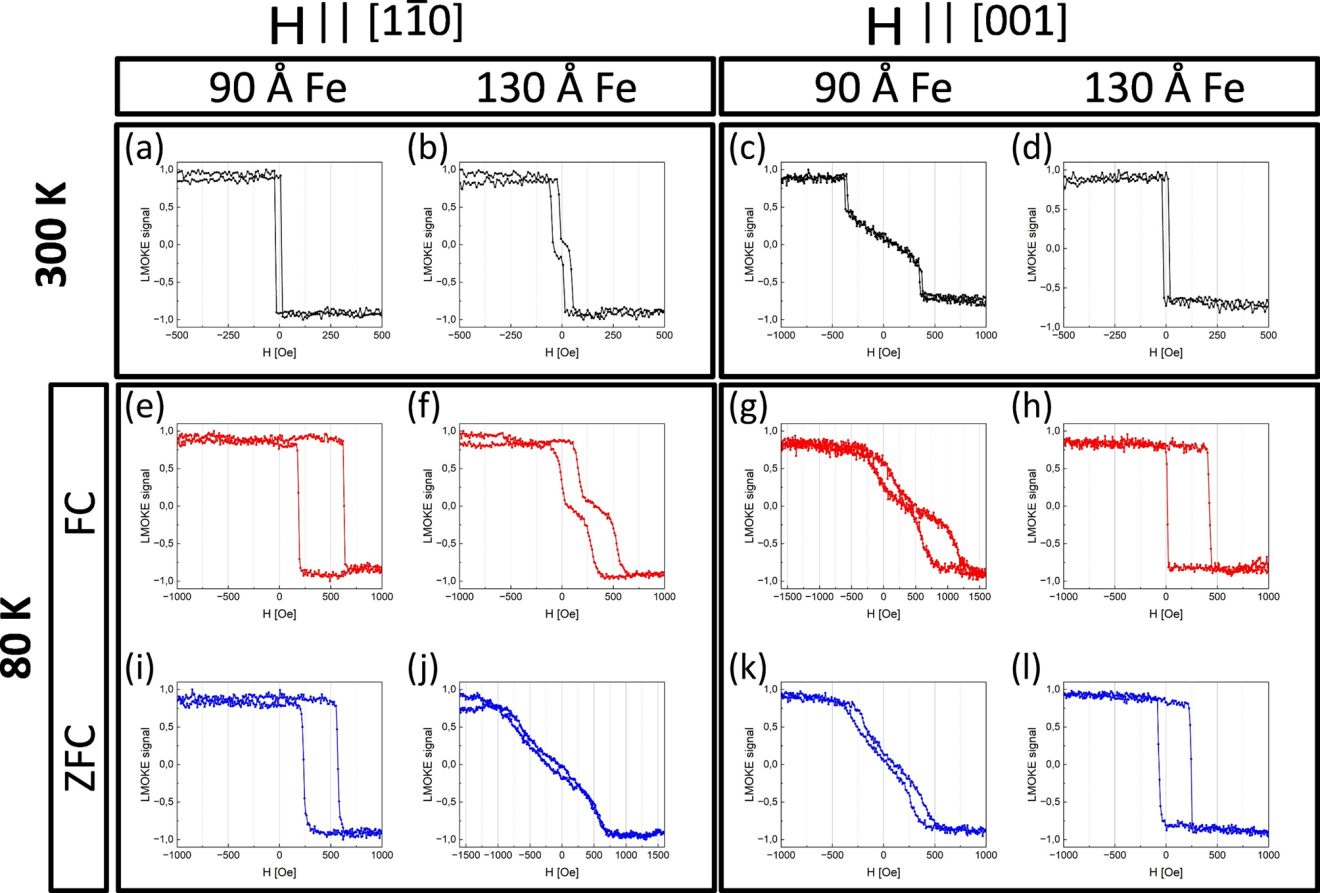
LMOKE measurements of CoO(111)/Fe(110) magnetic hysteresis loops, for 30 Å thick CoO overlayer, for different Fe thicknesses. Room temperature (**a**–**d**) and 80 K (**e**–**l**) measurements are shown for two in-plane orientations of external magnetic field, namely H || [$1\overline{1 }0$] and H || [001]. All FC loops (**e**–**h**) correspond to measurements after FC procedure in negative field along the same orientation as external magnetic field used for subsequent particular measurement. The ZFC loops (**i**–**l**) correspond to cooling without the external magnetic field after magnetizing the Fe layer using negative field along the particular measurement axis.

Magnetic properties of the samples were measured ex-situ using longitudinal magneto-optic Kerr effect (LMOKE) measurement setup. The dependence of the magnetic hysteresis loop shape and the corresponding shift field (H_EB_)on the relative orientation of the Fe layer easy axis and the magnetic field applied during the field cooling procedure was followed. In Fig. 2a-d room temperature LMOKE measurements of 30 Å CoO/90 Å Fe and 30 Å CoO/130 Å Fe sample regions are shown for two orthogonal longitudinal MOKE geometries. For H || [$1\overline{1 }0$] LMOKE geometry (Fig. 2a-b) the easy axis square hysteresis curve is measured for 30 Å CoO/90 Å Fe area, while a typical for Fe(110)/W(110) hard axis loop is observed for 30 Å CoO/130 Å Fe. For H || [001] (Fig. 2c,d) geometry, the situation is inverted. Those results confirm expected effect of thickness induced spin-reorientation transition (SRT) causing change of the easy axis of Fe from [$1\overline{1 }0$] to bulk-like [001] orientation. This phenomenon is well known and was in past reported for uncovered Fe(110) films^9–11^ as well as for those covered by non-magnetic^12^, ferromagnetic^13,14^ and more recently also antiferromagnetic overlayers^15–18^. Please note, that the hard axis loop in Fig. 2b is characterized by a very small anisotropy field, which means that for these particular thicknesses of CoO and Fe sublayers, Fe is very close to critical thickness of the SRT at 300 K. Magnetic hysteresis loops shown in Fig. 2e–h were measured after cooling the sample to 80 K in applied external, negative magnetic field of 1600 Oe. For those loops, called going forward as ‘field cooled’ (FC) loops, during cooling the sample was kept in negative external magnetic field parallel to the field direction during the subsequent LMOKE measurements. The aim of such FC experiments was to freeze interfacial CoO spins along the chosen axis irrespectively of Fe thickness. For the so called zero-field cooled (ZFC) loops (Fig. 2i–l), first the sample was magnetically saturated at room temperature by negative magnetic field along the particular measurement axis. Subsequently, the sample was cooled in its remanent state (H = 0).

All FC loops (e–h) exhibit negative EB, i.e. the loop is shifted towards positive values of the external magnetic field after cooling the sample in a negative magnetic field. For the CoO/90 Å Fe the H_EB_ is larger than for the thicker Fe sample area, which is naturally expected due to interfacial nature of EB effect which usually follows a H_EB_ ~ 1/d_Fe_ dependence. Additionally, the loop shift is similar for the same sample areas, as seen by comparing the magnetic hysteresis loops in Fig. 2 e and g or f and h, irrespectively of whether CoO spins were frozen along hard or easy Fe axis. As expected, for the ZFC loops (i-l) only the easy axis loops have non-zero H_EB_. The lack of the shift field for hard axis loops in Fig. 2j, k means that CoO spins in the corresponding sample areas are frozen along axis perpendicular to the respective LMOKE measurement axis.

**Fig. 3:**
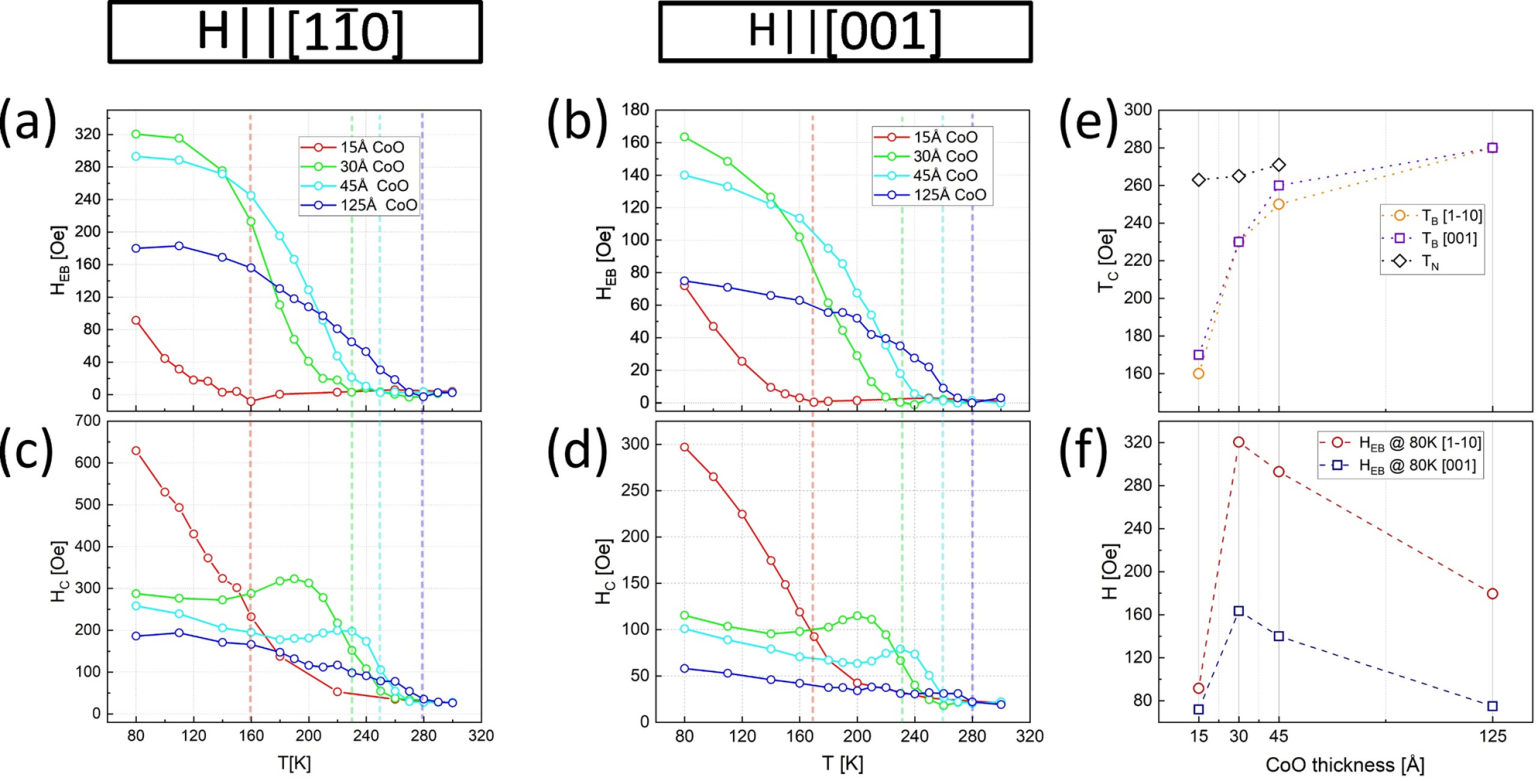
Temperature dependence of (**a**-**b**) shift field (H_EB_)and c-d) coercivity (H_C_) of magnetic hysteresis loops, with the T_B_ marked by dashed lines. Results are determined from LMOKE data for a field cooled sample for (**a**, **c**) 50 Å Fe step and (**b**, **d**) 120 Å Fe areas. Data acquired for (**a**, **c**) **H** || Fe[$1\overline{1 }0$] and (**b**, **d**) **H** || Fe[001], LMOKE geometries. CoO thickness dependence of (**e**) critical temperatures and (**f**) H_EB_ at 80 K.

The LMOKE technique was also used to measure the temperature dependence of easy axis magnetic hysteresis loops in the 80–300 K temperature range. For each hysteresis loop its coercivity (H_C_) and shift field (H_EB_) were determined, chosen representative hysteresis loops are shown in the Supplementary Material (Fig. S1). Equations used for H_EB_ and H_C_ determination, with a schematic easy and hard magnetization axis loops, can be found in Supplementary material (Fig. S2). The sample was FC, from 310 to 80 K, in a negative external magnetic field of 1600 Oe parallel to the field direction during LMOKE measurements. Because of the significant training effect, the measurements for 15 Å CoO thickness were done by repeated field cooling to each intermediate temperature from 310 K and measuring the first loop at given temperature. Coercive and shift field temperature dependencies, for different thicknesses of CoO overlayers are shown in Fig. 3a, b and c, d, respectively. Two selected Fe thicknesses were analysed, namely 50 Å and 120 Å, as measured along their respective easy magnetization axes **H** || [$1\overline{1 }0$] and **H** || [001] LMOKE geometries, see Fig. 3a, c and b, d, respectively. Please note, that the sample with 50 Å Fe area was chosen for those systematic temperature measurements as it exhibited the highest value of exchange bias which allows for more precise definition of the blocking temperature. Comparing to sample presented in Fig. 2, the behaviour of studied samples on 50 Å and 90 Å thick Fe, is quantitatively the same from the magnetic point of view. This is because the Fe thicknesses 50 Å and 90 Å both have the “thin” iron easy magnetization axis, i.e.^1–10^ which means “before” SRT.

**Fig. 4:**
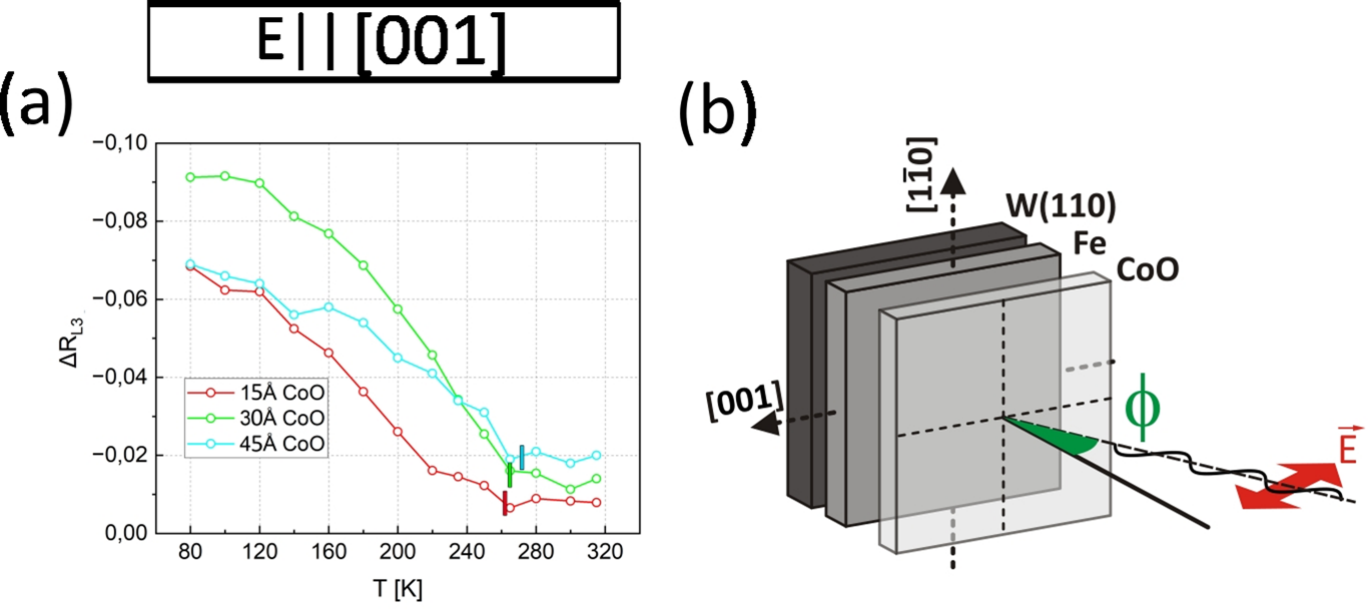
(**a**) Temperature dependence of ΔR_L3_ as derived from XAS spectra for CoO/120 Å Fe sample region. Vertical colour bars indicate T_N_ temperatures as determined from analysis presented in Fig. S5 of Supp. Material. (**b**) Schematics of the XMLD experiment geometry showing that the incoming X-ray beam electric field projection on the sample plane was parallel to Fe[001].

Data presented in Fig. 3a-b shows that the T_B_ strongly depends on CoO thickness, i.e. with decreasing the thickness of CoO layer the T_B_ decreases as well. Importantly, T_B_ is almost perfectly independent of the axis along which CoO moments are oriented. Clearly, for given CoO thickness T_B_ is the same for **H** || [1-10] (Fig. 3a) and **H** || [001] (Fig. 3b) data. This can also be seen in Fig. 3e, where critical temperatures are plotted as a function of CoO thickness. From Fig. 3f it can also be observed that for the given Fe thickness, the highest H_EB_ is present at the 30 Å thick CoO, which means that instead of continuous rise of H_EB_ with CoO thickness, a non-monotonic dependence with a clear maximum of H_EB_(d_CoO_) is observed. Such behaviour was often reported in the past^19^ and usually the initial exchange bias enhancement is ascribed to stronger interfacial coupling between the AFM and FM layers and increased stability of antiferromagnetic spins. However, at a certain AFM thickness, antiferromagnetic domain formation and bulk contributions to AFM magnetic anisotropy become more significant, leading to a decrease of the exchange bias as the AFM layer continues to thicken. Also important is the previously mentioned monotonic T_B_ vs d_CoO_ dependence. It is in some cases interpreted in terms of the so called finite-size effects^20^. Such phenomena result from strength of spin–spin interactions between inner and surface AFM atoms, which can be modified due to reduced number of neighbours at the surface. The resulting increase of surface or interface lattice vibration explains why for thinner layers, with larger surface/volume ratios, their critical temperatures are often smaller than for the bulk material. To test such hypothesis, XMLD measurements were performed, results of which are presented in Fig. 4.

In order to directly probe the T_N_ of CoO layers instead of blocking temperatures T_B_ of CoO/Fe bilayers, XMLD^21–23^ technique was employed. The inset in Fig. 4b shows an illustration of the XMLD geometry. A signature of the antiferromagnetic order in CoO can be directly followed from the comparison of the X-ray Absorption Spectroscopy (XAS) spectra acquired at two specific XMLD geometries, namely normal and grazing incidence geometries for which the polar angles are φ = 0° and φ = 60°, respectively, see comparison and formula used to calculate ΔR_L3_ in Supplementary material, Fig. S4. Following the analysis of angle resolved XMLD measurements presented in^16^, we conclude that CoO spins are oriented within the sample CoO(111) || Fe(110) plane. From Fig. 4a, the decrease of ΔR_L3_ absolute value is observed for all thicknesses of CoO up to ~ 265 K temperature. Above this temperature, XMLD as probed by ΔR_L3_, becomes constant with temperature, which marks the Néel temperature T_N_^24^ which in our sample is independent of CoO thickness. We do not present XMLD data for thickest (125 Å) studied CoO overlayer for which its topmost surface layer (~ 10–30 Å thick) significantly deviates from stoichiometric CoO and makes the reliable analysis of XAS intensity peaks impossible. The observed deviation from CoO stoichiometry could potentially influence H_EB_ for thicker AFM films. While we cannot completely exclude such possibility, results shown in Fig. 3f suggest that those two effects are rather independent. Clearly, H_EB_ starts to decrease already for 45 Å thick CoO, where the AFM layer stoichiometry is not deviated at all.

The XMLD (Fig. 4a) results together with the LMOKE measurements (Fig. 3a-b) indicate that the CoO thickness dependence of T_B_ cannot be the result of previously mentioned finite-size effects. This is because in contrast to the blocking temperature T_B_, the Néel temperature T_N_ of CoO films are practically independent on the thickness of AFM layer. An alternative mechanism relies on the existence of antiferromagnetic moments that become rotatable rather than frozen, at temperatures lower than T_N_. The proportion of rotatable and frozen AFM spins is CoO thickness dependent, which explains why T_B_ is reduced with decreasing d_CoO_. The existence of such type of AFM spins was proposed for polycrystalline systems and applicability of the corresponding theoretical framework was suggested also for monocrystalline samples in the pioneering work by Stilles^25^. In such model, the AFM domains are expected to be frozen at low temperature but as the sample approaches the T_B_ some of them start to be rotatable, which means that their orientation can change and follow the orientation of neighbouring Fe spins. Such rotation of AFM spins can be triggered either by magnetization reversal of Fe or by spontaneous reorientation of Fe spins induced for example by increasing temperature. At temperature range where all of the AFM spins are unfrozen, but AFM layer is still magnetically ordered, i.e. at T_B_ < T < T_N_, magnetic moments of AFM follow any reorientation of FM magnetization and as a consequence unidirectional magnetic anisotropy is diminished which makes the H_EB_ ~ 0. This however does not necessarily mean that EB effect is fully suppressed, as still the interaction with AFM magnetic moments can influence the Fe magnetization reversal. In Fig. 3c-d temperature dependencies of coercive field H_C_ are shown for 30 Å and 45 Å CoO thicknesses. At temperatures around 30 K below the respective T_B_, a pronounced coercivity bump is observed for easy axis loops measured in both LMOKE geometries. Such coercivity enhancement in the vicinity of blocking temperature of AFM/FM bilayer was theoretically predicted^26^ and later also experimentally documented^27–29^. The existence of coercivity peak ~ T_B_ supports the idea of rotatable AFM domains that are thermally activated. As a result, during Fe magnetization reversal, larger external magnetic field is required to reorient ferromagnetic moments because in these thermally unstable regions also antiferromagnetic rotatable spins are dragged, at additional energy cost.

**Fig. 5:**
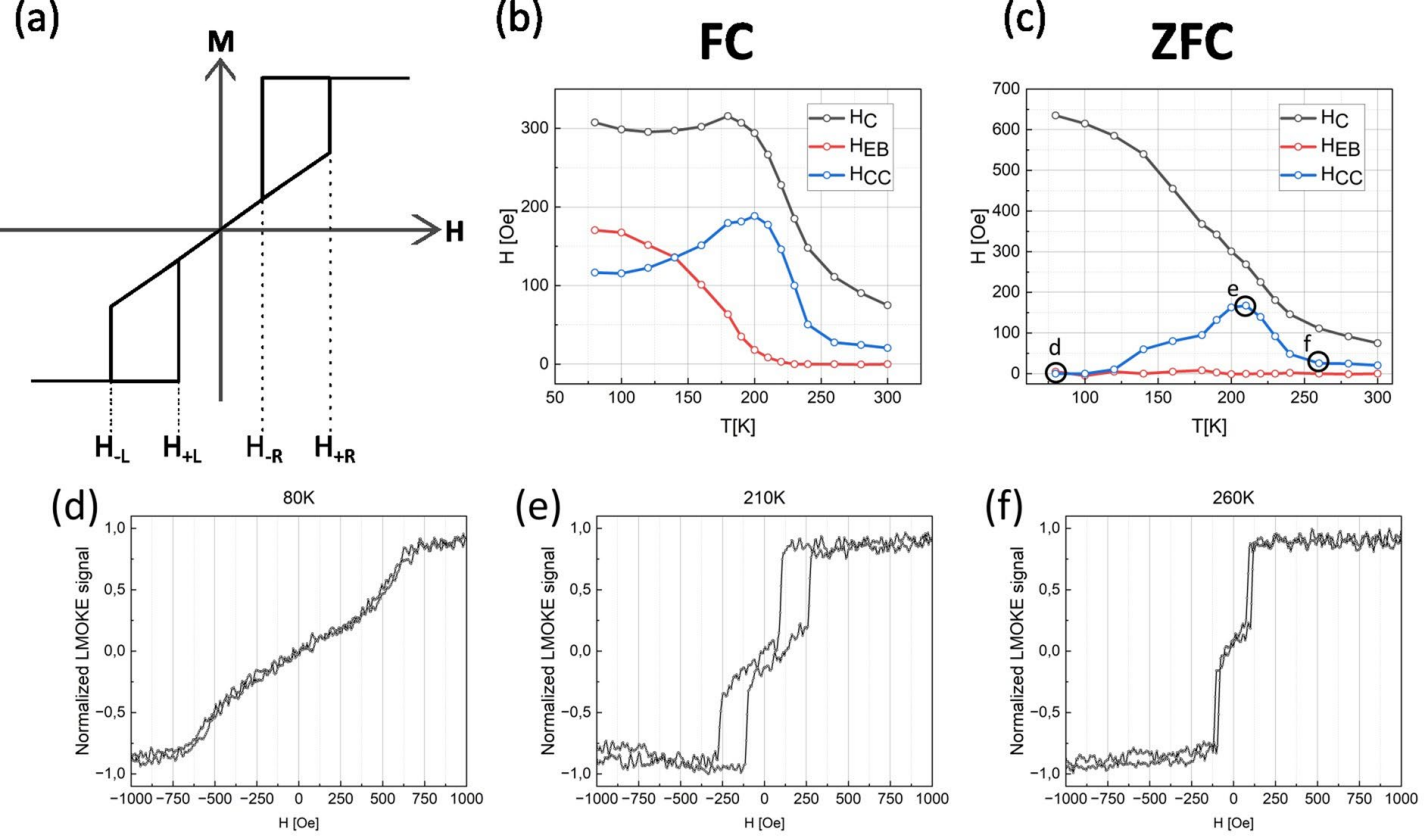
(**a**) Schematic definition of parameters used to calculate H_CC_. (**b**–**c**) Temperature dependence of the shift field (H_EB_,) coercivity (H_C_) and H_CC_ parameters as determined from LMOKE data acquired for H || Fe[$1\overline{1 }0$] geometry, after (**b**) field cooling (FC) and (**c**) cooling without external magnetic field (ZFC). Data for 30 Å CoO on 120 Å thick Fe sample region is shown. In (**d**–**f**) exemplary ZFC magnetic hysteresis loops are shown.

In addition to easy axis magnetic hysteresis loops, the temperature dependent LMOKE measurements were also performed for hard axis of 30 Å CoO/120 Å Fe sample. In this case, the choice of 30 Å CoO/120 Å Fe sample was due to optimal properties of chosen sample area which allows to demonstrate specific effects in the most clear way. The 30 Å CoO sample area was chosen as it exhibits the highest EB at 80 K. Furthermore, the 120 Å Fe sample area and^1–10^ LMOKE geometry was chosen as such relatively large thickness of the ferromagnetic layer allows for good quality measurements of the hard axis hysteresis loops. The measurements were done after both FC and ZFC cooling to 80 K, as described in the previous section. All ZFC magnetic hysteresis loops are fully symmetric with respect to zero field axis, which means H_EB_ = 0. Also, their coercivity temperature dependence does not show characteristic peak of H_C_ around blocking temperature. However, still the influence of AFM spins on magnetization reversal can be noticed. In order to quantify this effect we define a H_CC_ parameter, formula for which is presented below:

1 $${{\boldsymbol{H}}}_{{\boldsymbol{C}}{\boldsymbol{C}}}=\frac{\left|{{\boldsymbol{H}}}_{+{\boldsymbol{L}}}{-{\boldsymbol{H}}}_{-{\boldsymbol{L}}}\right|+\left|{{\boldsymbol{H}}}_{+{\boldsymbol{R}}}-{{\boldsymbol{H}}}_{-{\boldsymbol{R}}}\right|}{2},$$

where H_–L_, H_+L_, H_−R_ and H_+R_ parameters are defined on the schematic ZFC hard axis hysteresis loop shown in Fig. 5a. The results of the analysis of both FC and ZFC loops along with selected ZFC loops are presented in Fig. 4b–f.

The H_C_(T) curve for the FC procedure (Fig. 5b) exhibits similar H_C_ bump as the one for easy axis loops for the same sample region (Fig. 3c) and as already mentioned the corresponding ZFC dependence (Fig. 5c) do not exhibit the peak of coercivity ~ T_B_. However, the ZFC loops significantly change their shape with temperature. Specifically, two characteristic magnetization jumps observed during Fe reversal along hard axis, are well known to display hysteretic behaviour^30^. Magnetization, in general can rapidly switch for different external magnetic field values within ascending and descending branches of given half of the hysteresis loop. In principle, our H_CC_ parameter probes the width of such ‘hysteresis window’ which apparently is strongly temperature dependent, see in Fig. 5 d-f. Both the FC and ZFC series of measurements (Fig. 5 b-c) exhibit H_CC_ bump at similar temperature, which is also comparable to the temperature of the H_C_ bump at the same 30 Å CoO/120 Å sample area but measured along the Fe[001] easy axis (Fig. 3c). Both H_CC_ for hard axis loops and H_C_ for easy axis loops are parameters of hysteresis of the magnetization reversal and the enhancement of both these parameters is observed at similar temperatures. We therefore conclude that the same mechanism of un-freezing of AFM magnetic moments is reflected in temperature dependencies of both H_C_ and H_CC_.

**Fig. 6:**
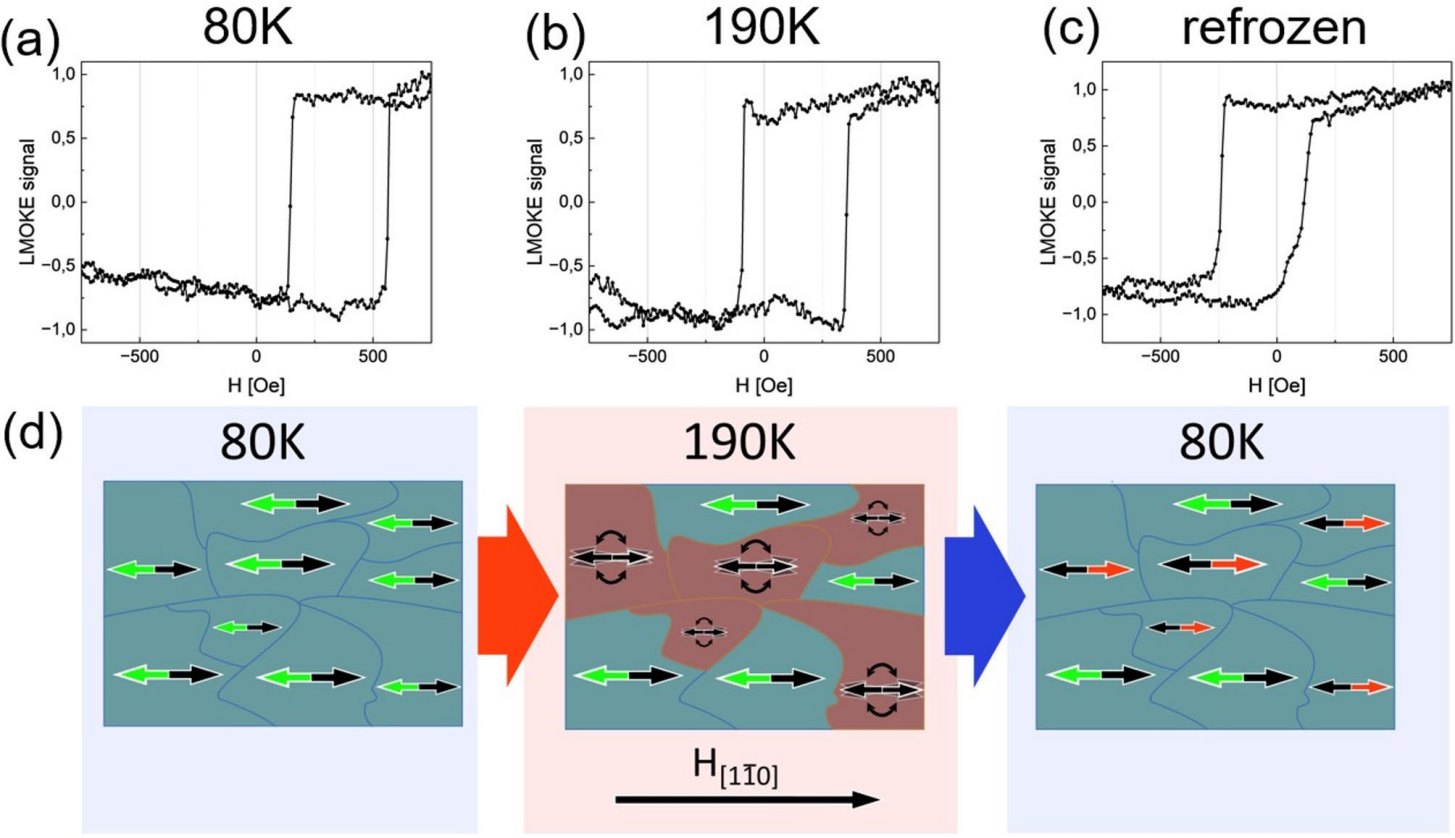
(**a**–**c**) Measured hysteresis loops of 30 Å CoO/ 80 Å Fe sample area for **H** || Fe[$1\overline{1 }0$] LMOKE geometry. a) Hysteresis loop at 80 K after FC in negative external magnetic field, (**b**) after heating to 190 K and (**c**) after FC re-cooling to 80 K with positive external magnetic field applied. (**d**) Schematics of unfreezing and refreezing of AFM domains. Green and red arrows schematically mark the direction (to the left and right) of interfacial AFM spins responsible for particular H_EB_ sign (positive and negative, respectively).

The occurrence of such thermally activated, rotatable AFM magnetic moments should be detectable, providing the appropriate cooling and measurement procedure is employed. Indeed, magnetic hysteresis loops strongly support the existence of small AFM/FM domains or regions, as explained in details in the following. The procedure starts with the FC cooling of the sample from 310 to 80 K, with the negative external magnetic field **H** || Fe[$1\overline{1 }0$] of 1600 Oe. At 80 K the easy-like magnetic hysteresis loop with positive H_EB_ is measured for 30 Å CoO/ 80 Å Fe sample area, see Fig. 6a. Next, the sample is heated up to 190 K, close to the T_B_ but still significantly below T_N_, where similar, although less exchange biased magnetic hysteresis loop is acquired (Fig. 6b). From 190 K, the sample is again FC cooled, this time with positive external magnetic field, of the same magnitude, i.e. with **H** || Fe[$1\overline{1 }0$]. At 80 K, the easy-like magnetic hysteresis loop but with negative shift field is documented. In Fig. 6d we schematically depict proposed behaviour of AFM domains during such experiment. When the sample is field cooled with the negative external magnetic field **H** || Fe[$1\overline{1 }0$], all of the AFM spins are aligned along the Fe[$1\overline{1 }0$], which is the easy axis of the 30 Å CoO/80 Å Fe sample area. The interfacial AFM spins responsible for positive H_EB_ in this sample state are marked by green arrow in Fig. 6d. Once the sample is heated close to the blocking temperature, some of the AFM magnetic moments become rotatable, see Fig. 6d scheme for 190 K sample state. During the re-cooling of the sample, this time with the assistance of positive external magnetic field, the rotatable AFM spins become frozen along Fe[$1\overline{1 }0$] which is antiparallel to Fe[$1\overline{1 }0$] direction. Finally, all of the AFM domains are again frozen at low temperature, all along the same in-plane axis but each with its own local direction of interfacial AFM spins (green and red arrows in Fig. 6d) responsible for EB effect. Note, that in this final state, measured LMOKE signal (Fig. 6c) results from interaction of Fe magnetization with those two types of AFM domains. Importantly, acquired magnetic hysteresis curve (Fig. 6c) is not a simple algebraic sum of two easy-like loops with opposite (in our notation positive and negative) H_EB_. Such hypothetical loop would be a result of simple spatial average of LMOKE signal from two types of independent AFM/FM domains. Magnetic hysteresis loop presented in Fig. 6c indicates another scenario in which Fe magnetization of the probed sample region interacts with both AFM (green and red) domains. As a result, a single easy-like loop with particular H_EB_ sign is observed. From small but negative H_EB_ value in Fig. 6c, we conclude ‘red’ AFM spins to be slightly more represented over those marked as ‘green’, in this particular sample state obtained in our experiment after re-cooling to 80 K. If we assume a simple relation between the amount of magnetic moments rotated in the experiment and the H_EB_ value, the proportion of the magnetic moments successfully rotated can be estimated using the following formula:

2 $${{\boldsymbol{H}}}_{{\boldsymbol{E}}{\boldsymbol{B}}}={{\boldsymbol{H}}}_{{\boldsymbol{E}}{\boldsymbol{B}}\_{\boldsymbol{M}}{\boldsymbol{A}}{\boldsymbol{X}}}\left(1-{2{\boldsymbol{S}}}_{{\boldsymbol{r}}}\right)$$

In the above formula (2), H_EB_ is exchange bias value, with a particular sign dependent on whether the loop is shifted in the positive or the negative direction of the field, H_EB_MAX_ is the maximum exchange bias measured before the sample heating, and S_r_ is the amount of magnetic moments rotated, S_r_ = 1 meaning all and S_r_ = 0 meaning no spins rotated. From the formula (2) we can estimate that roughly.

 ~ 58% of the magnetic moments were rotated in our experiment, therefore such amount of magnetic moments become rotatable at 190 K.

**Fig. 7:**
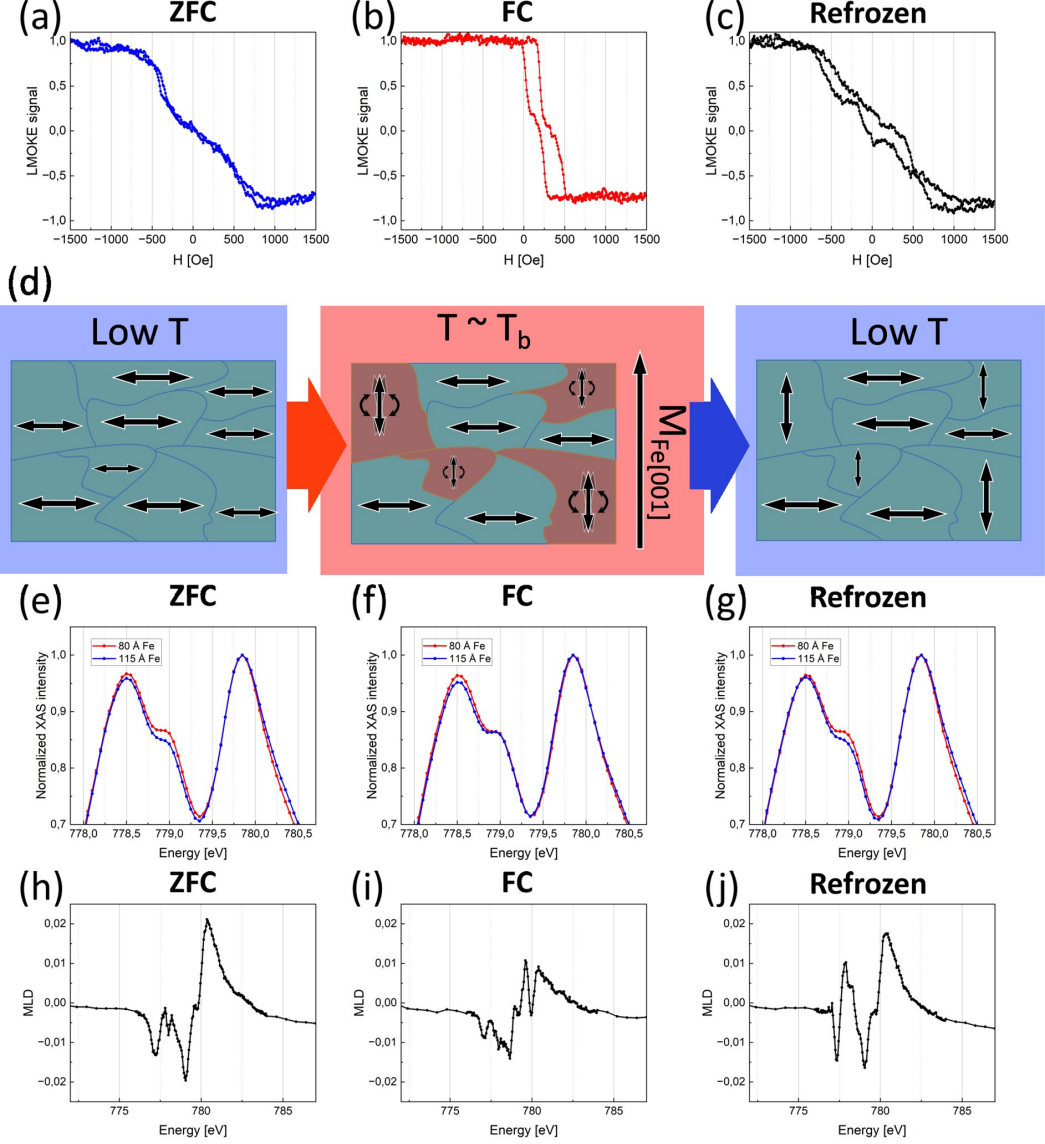
(**a**–**c**) Measured hysteresis loops of 30 Å CoO/ 130 Å Fe sample area at 80 K for **H** || Fe[$1\overline{1 }0$] axis after ZFC and FC cooling and after refreezing from 230 K. (**d**) Schematics of unfreezing and refreezing of AFM domains. XAS and MLD spectra for 25 Å CoO/115 Å Fe and 25 Å CoO/80 Å Fe sample regions measured at 80 K after (**e**, **h**) ZFC, (**f**, **i**) FC and (**g**, **j**) after ‘refreezing’ from 230 K.

Similar experiment can performed for hard axis loops. In this case, the aim is to document unfreezing and refreezing of 90 degree (instead of 180 degree) rotated interfacial AFM spins. In this case, employed procedure starts with the ZFC cooling of the sample from 310 to 80 K. Subsequently, the hard axis, exchange bias free loop is measured at 80 K, see example for the 30 Å CoO/ 130 Å Fe sample measured in **H** || Fe[$1\overline{1 }0$] LMOKE geometry, Fig. 7a. Next, the sample was field cooled, with the negative external field of 1600 Oe along the Fe[$1\overline{1 }0$] axis, from 310 to 80 K and the FC magnetic hysteresis loop was measured, see Fig. 7b. As expected, hard-like loop with significant H_EB_ is observed. After this measurement, the sample was heated to 230 K, close to the T_B_ but still significantly below T_N_, and cooled back down to 80 K, without the assistance of external magnetic field. In this final state, the ‘refrozen’ loop was measured at 80 K, result is plotted by solid line in Fig. 7c.

In Fig. 7d we schematically depict proposed behaviour of AFM domains during the experiment. When the sample is field cooled with the external magnetic field **H** || Fe[$1\overline{1 }0$] all of the AFM spins are aligned along the Fe[$1\overline{1 }0$], which is the hard axis of the 30 Å CoO/130 Å Fe and 25 Å CoO/115 Å Fe sample area. Once the sample is heated close to the blocking temperature some of the AFM magnetic moments become rotatable and, because of the lack of the external magnetic field, align themselves with the direction of magnetization of Fe, i.e. along [001] axis. During the re-cooling of the sample the rotated AFM domains remain in their new orientation along Fe[001] while the remaining domains (that were still not unfrozen) stay magnetized along the original Fe[$1\overline{1 }0$] direction. Once all of the AFM domains are again frozen at low temperature, each along its own local axis, we expect the LMOKE signal to result from Fe magnetization interaction with both AFM domain populations. The measured ‘refrozen’ hysteresis loop from Fig. 7c seems to confirm these expectations. Clearly, the magnetization jumps from the FC loop can be visible in it, albeit shifted slightly, while the general shape and saturation field is that of the ZFC loop. In our opinion, this is indirect but strong evidence for the existence of two types of AFM/FM domains frozen along two orthogonal in-plane axes. We attempt to estimate the proportion of the magnetic moments that were rotated using the modified formula (2) which accounts for the fact that now the rotated magnetic moments have no direct influence on the magnitude of the H_EB_. The modified formula is as follows:

3 $${{\boldsymbol{H}}}_{{\boldsymbol{E}}{\boldsymbol{B}}}={{\boldsymbol{H}}}_{{\boldsymbol{E}}{\boldsymbol{B}}\_{\boldsymbol{M}}{\boldsymbol{A}}{\boldsymbol{X}}}\left(1-{{\boldsymbol{S}}}_{{\boldsymbol{r}}}\right),$$

Very rough estimation of the amount of magnetic moments calculated from the formula (3) is ~ 94%. This value is much less reliable as compared to analysis of Fig. 6 results because of the irregular shape of the “refrozen” loop which makes its analysis less straightforward. Direct proof, for the existence of the two types of AFM/FM domains frozen along two axes, can be obtained using XMLD technique, however experimental implementation is difficult. Ideally, for given Fe thickness, orientation of CoO spins should be probed by XMLD and compared for remanent and ‘in-field’ sample states. The later one means that in-plane magnetic field should permanently be applied during XAS spectrum measurement. The difference of such two spectra would be a fingerprint of rotatable AFM spins appearance or absence. Unfortunately, at the PIRX end-station the in-plane component of external magnetic field is non-zero only in grazing incidence geometry of XAS experiment. In such case, in contrast to normal incidence geometry, the sensitivity to in-plane orientation of CoO spins is almost fully suppressed which makes such experiment technically not feasible. With the help comes the thickness induced in-plane SRT effect in Fe layers on W. In such solution Fe layer plays a built-in external magnetic field role, i.e. Fe makes the rotatable spins (if present) of CoO to orient along its magnetization. At the same time, the XAS spectrum in normal incidence geometry can be recorded. Two orthogonal in-plane orientations of Fe and CoO magnetic moments can be stabilized by means of the mentioned thickness induced SRT. In Fig. 7e–g, comparison of XMLD spectra acquired at 80 K for two Fe thicknesses is presented. Specifically, for 80 Å and 115 Å thick Fe layers their magnetization spontaneously orients along^1–10^ and [001] directions, respectively. Due to interfacial nature of the effect, especially for (111) oriented CoO films, the in-plane XMLD effect is usually very small. For this reason, the presented spectra are magnified around the photon energy ~ 779 eV, at which the highest change of XAS intensity is induced by rotation of CoO spins. Small, but noticeable change of XAS intensity peak at this energy can be followed depending on the state of the sample at 80 K. For ZFC state (Fig. 7e), two spectra are different because the CoO spins are frozen along orthogonal in-plane orientations, as dictated by the remanent states of 80 Å and 115 Å Fe regions during cooling of the sample. Such difference (XMLD) vanishes after the FC procedure, Fig. 7f; external magnetic field applied during cooling makes CoO spins to be frozen along its direction, independently on the thickness of Fe. Then the sample got heated up to 230 K, which is below the T_N_ and in the vicinity of T_B_, where we expect most of the CoO spins to be rotatable and therefore susceptible to the Fe magnetization. After refreezing from 230 to 80 K (Fig. 7 g), again the orthogonal configuration of AFM spins on the sample is restored. The presented differences in the XMLD signals are quite subtle which can raise the question concerning their statistical significance. All measured at the PIRX beamline XAS spectra have very low noise-to-signal ratio and for this reason presented XMLD differences, although very small, are indeed statistically significant and fully reproducible. The error bars in spectra shown in Fig. 7e-g are comparable in height to the size of the symbols and the line width on the graph. Importantly, results presented in Figs. 6 and 7 are reproducible, as confirmed by similar experiments performed on other CoO thicknesses and at slightly different temperatures.

## Conclusions

We used thickness induced SRT in CoO(111)/Fe(110) epitaxial bilayers as a tool to reorient and probe to detect the orientation of frozen and rotatable antiferromagnetic CoO spins. By combining the LMOKE results with the XMLD measurements we show that the Neel and blocking temperatures are different in CoO(111)/Fe(110) system. While the T_N_ is the same for all measured CoO thicknesses, the T_B_ strongly depends on the thickness of CoO layer which can be explained by the thermal stability of rotatable antiferromagnetic moments that goes beyond classical finite-size effects. Our results provide evidence for the existence of AFM magnetic moments, larger proportion of which becomes rotatable by the FM layer as temperature increases. While those regions or domains retain AFM ordering, once sufficient amount of them becomes rotatable, the exchange bias shift field vanishes. Measurements show that such rotatable spins can be refrozen in a new orientation, providing the temperature is temporarily increased to vicinity of T_B_ but below T_N_. Such interfacial exchange coupling and magnetic anisotropy engineering can potentially be useful for future spin valves and spin–orbit torque ideas in FM/AFM systems.

## Methods

### Samples preparation

Series of samples were made on an atomically clean W(110) single crystal. Epitaxial Fe(110) films of different thicknesses were grown at room temperature, as macroscopic steps, using molecular beam epitaxy (MBE), followed by annealing at 700 K. This produced high-quality Fe films with an atomically smooth (110) surface. Next, CoO(111) adlayer steps perpendicular to the Fe(110) steps were grown, resulting in a checkerboard-like architecture, by reactive deposition of Co in an O_2_ atmosphere (partial pressure of 1 × 10^–6^ mbar) at room temperature. Prior to the reactive deposition of CoO, 2 Å–thick metallic Co was grown on the Fe surface in order to minimize the oxidation of Fe layer. The thicknesses of the deposited layers were measured in-operando using quartz crystal microbalance. During the MBE deposition successive parts of the sample are covered from the molecular beam by the shutter placed in front and close to the sample surface. Small shadow wedge at the border between each two macroscopic steps exists but because of its microscopic size (of the order of 10 µm) shadowing and averaging effects are irrelevant for measurements. The shortest size of checkerboard-like sample areas is 0.5 mm.

### Structural properties

The low-energy electron diffraction (LEED) technique was used to study the surface structure of the Fe(110) and CoO(111)/Fe(110) film. Symmetry of diffraction images observed by LEED confirm the epitaxial (110) oriented Fe films and (111) oriented CoO overlayers.

### Magnetic properties

The magnetic properties of the CoO/Fe(110) system were studied ex situ using longitudinal magneto-optic Kerr effect (LMOKE) combined with liquid nitrogen cryostat. Temperature dependence of LMOKE magnetic hysteresis loops M(H) was followed for two complementary LMOKE geometries, namely with the external magnetic field H applied either along the Fe[$1\overline{1 }0$] or Fe[001] in-plane direction.

The X-ray Absorption Spectroscopy (XAS) spectra were ex-situ measured at the PIRX end station of National Synchrotron Radiation Centre Solaris in Kraków^31,32^. X-ray magnetic linear dichroism (XMLD) was measured on the L_3_ absorption edge of Co. In case of AFM CoO, XMLD magnitude is defined by the so called R_L3_ ratio of the XAS intensity at photon energy of 777.2 eV divided by the intensity at 779.8 eV. A signature of the antiferromagnetic order in CoO can be directly followed from the comparison of the XAS spectra acquired at two specific XMLD geometries, namely normal and grazing incidence geometries for which the polar angle is φ = 0° and φ = 60°, respectively. Next, a differential R_L3_ is determined as ΔR_L3_ = R_L3_(φ = 0°)—R_L3_(φ = 60°). In our experimental geometry, the linearly polarized X-ray beam was used and its electric field vector **E** projection onto the sample plane was parallel to the Fe[001] in-plane direction, **E**_**in-plane**_ || Fe[001].

All measurements probes are significantly smaller than shortest size of checkerboard-like sample areas, i.e. the spot of the MOKE laser is ~ 0.25 mm and in case of XAS measurements the spot of the X-ray beam on the sample depends on the geometry and settings of the beamline optics but does not exceed ~ 200 µm.

## Supplementary Information

Below is the link to the electronic supplementary material.

Supplementary Material 1

## Data Availability

The data that support the findings of this study are available from the corresponding author upon reasonable request.
