# Supplementary material for: Freezing and unfreezing of antiferromagnetic spins in CoO(111) epitaxial films on a ferromagnetic support
Source: Sci Rep. 2025 Dec 19;16:2809. doi: 10.1038/s41598-025-32592-9 (PMC12824345; doi:10.1038/s41598-025-32592-9)
Supplement: Supplementary file 1 — Supplementary Material 1 [file 41598_2025_32592_MOESM1_ESM.docx]

**Supplementary material for:**

**Freezing and unfreezing of antiferromagnetic spins in CoO(111) epitaxial films on a ferromagnetic support.**

A. Kwiatkowski^1^, M. Szpytma^2,3^, E. Świerkosz^1^, E. Oleś^1^ , P. Dróżdż^1^ , A. Kozioł-Rachwał^1^, M. Zając^3^, E. Partyka-Jankowska^3^, T. Ślęzak^1^, M. Ślęzak^1*^

^1^ AGH University of Krakow

^2^ Elettra-Sincrotrone Trieste

^3^ SOLARIS National Synchrotron Radiation Centre, Jagiellonian University

*mislezak@agh.edu.pl

For both CoO and Fe sublayers, various thicknesses are presented across the whole manuscript. However, we are confident that the presented study is fully consistent from the point of view of magnetic and structural properties of the studied samples. Qualitatively, samples with Fe thickness 50, 80 or 90 Å magnetically behave in the same way as they all have the “thin” iron easy magnetization axis, i.e. along Fe[1-10], which means “before” SRT. The same concerns the case of 115, 120, 130 and 200 Å thicknesses, which all are “above” SRT and spontaneously orient their magnetization along [001] direction. The choice of particular thickness of Fe or CoO was in each particular case dictated by the magnitude of EB, quality of measured MOKE loops or desired in-plane easy axis orientation (along Fe[1-10] or Fe[001]) of ferromagnetic or antiferromagnetic component.


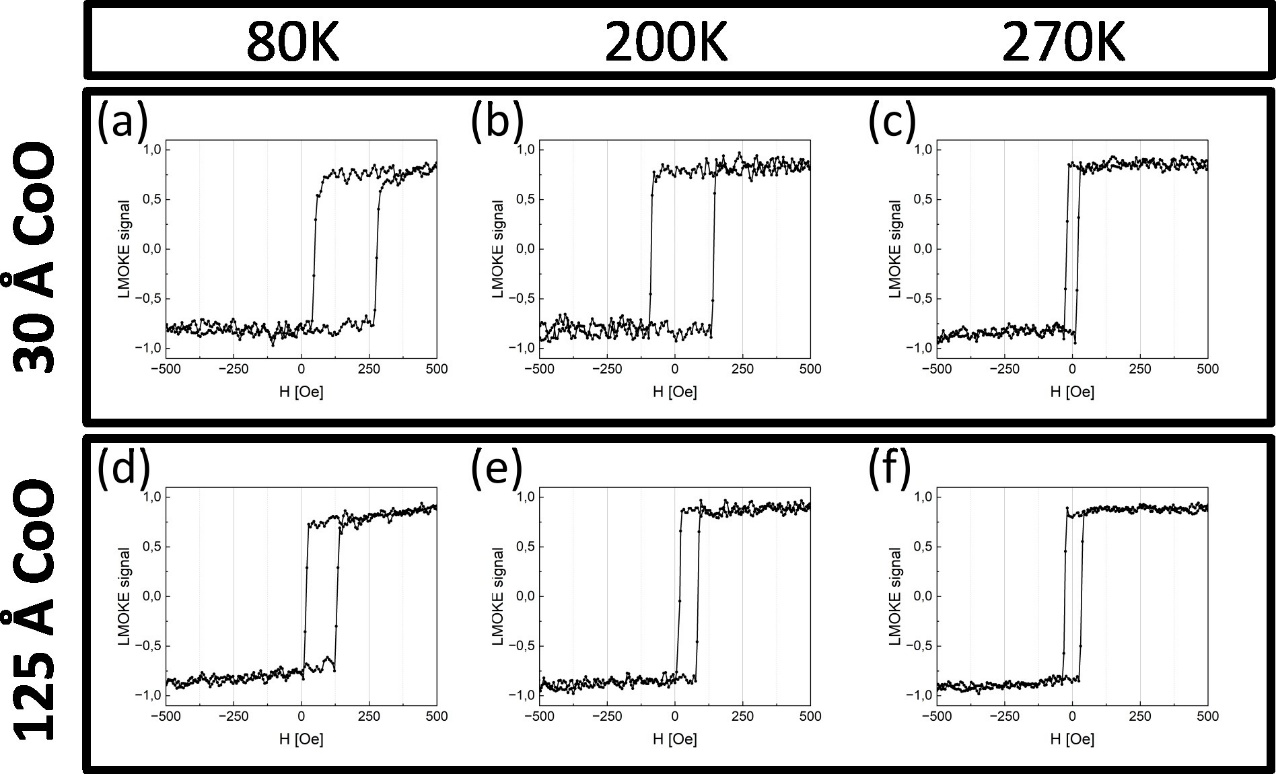


Fig. S1 Measured LMOKE hysteresis loops on 120 Å Fe for (a-c) 30 Å CoO and (d-f) 125 Å CoO sample area at different temperatures after FC in negative external magnetic field. All presented loops were measured in and **H** || [001] LMOKE geometry.


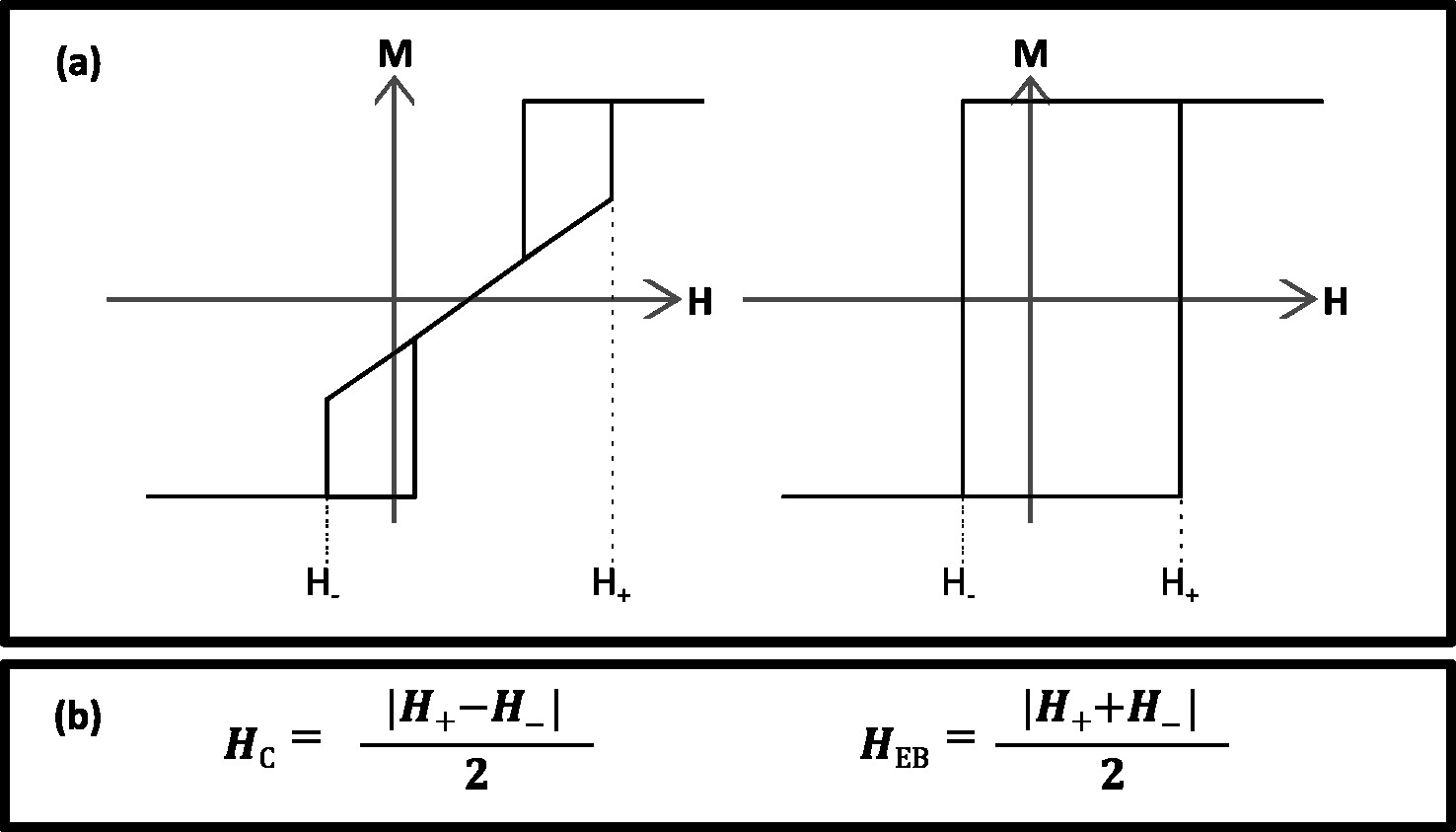


Fig. S2 a) Schematics of hard axis and easy axis Fe hysteresis loop with important parameters highlighted. b) the equation used to determine the H_C_ and H_EB_ parameters of all measured hysteresis loops.


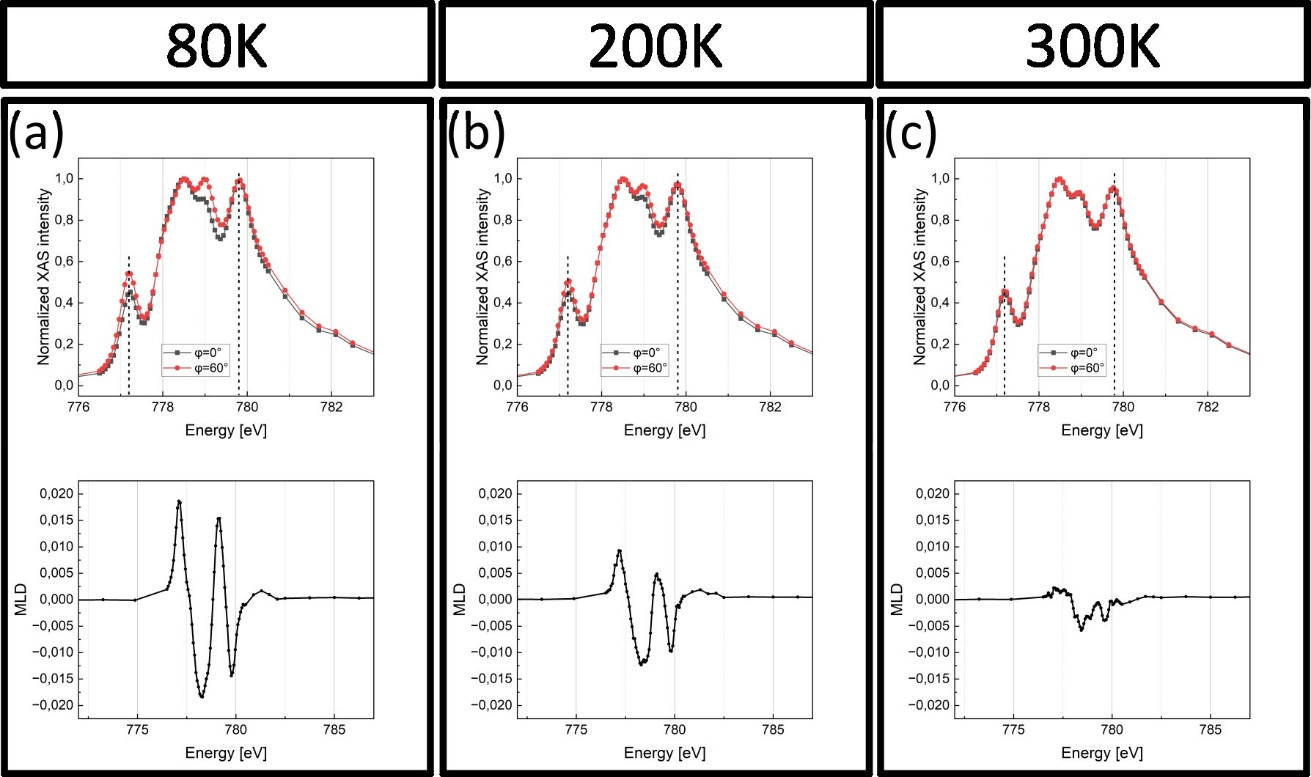


**Fig. S3** XAS spectra acquired in normal (black) and grazing (red) incidence geometry around L_3_ absorption edge of Co, with linearly polarized X-rays and their MLD spectra. Presented data was obtained at a) 80 K, b) 200K, c) 300K for 30 Å CoO/120 Å Fe. First and fourth of the characteristic intensity peaks used for ΔR_L3_ ratio definition are marked by dashed, vertical lines.


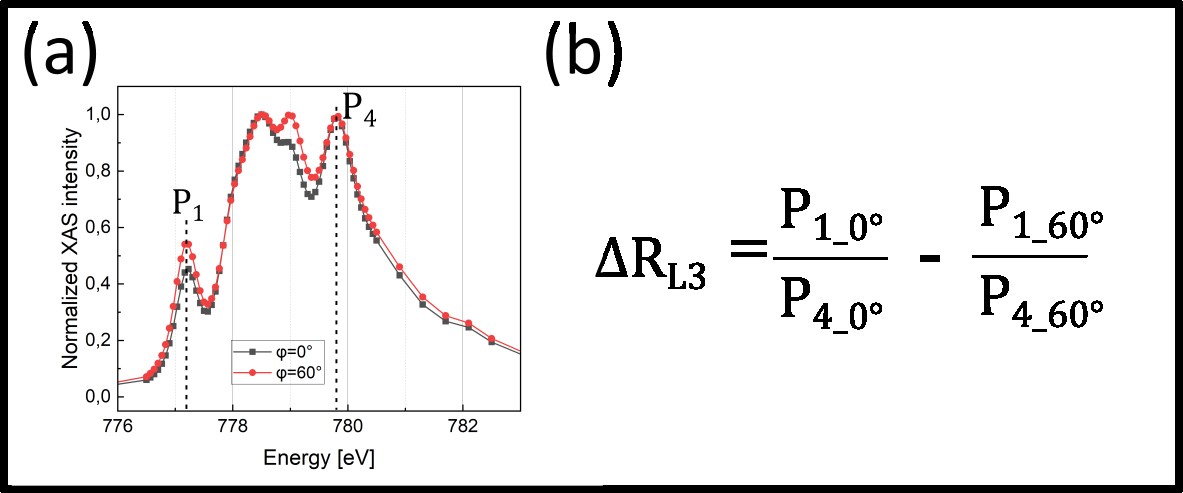


**Fig. S4 a)** XAS spectra acquired in normal (black) and grazing (red) incidence geometry around L_3_ absorption edge of Co, with linearly polarized X-rays at 80 K for 30 Å CoO/120 Å Fe. First and fourth of the characteristic intensity peaks used for ΔR_L3_ ratio definition are marked by dashed, vertical lines called P_1_ and P_4_, respectively. b) The formula used to calculate ΔR_L3_, with the polar angle index defining in which geometry the spectrum was measured.


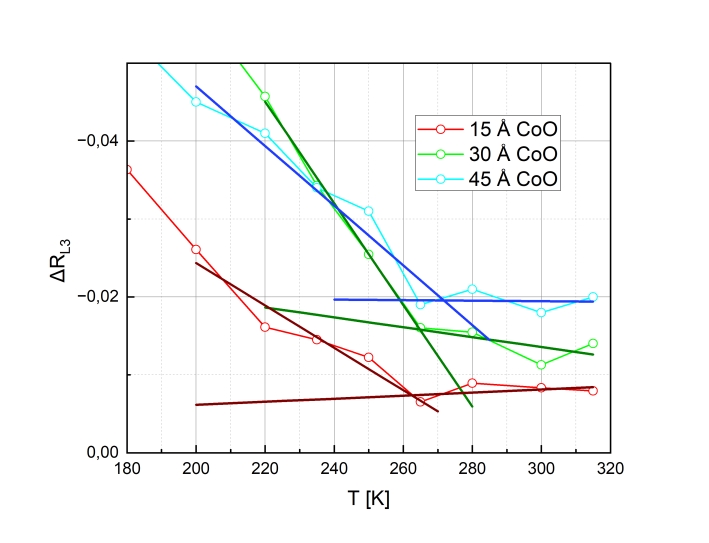


**Fig. S5**. Determination of the T_N_ temperatures. The intersection of two regression lines is usually used to determine critical temperature in a more quantitative way. First of these lines is fitted to the non-zero slope region in the temperature range just below the critical temperature. The second line is fitted to zero-slope above expected T_N_ where ΔR_L3_ is constant or nearly constant.
